# Real-World Testing of a Clinical Strategy to Start Early Peritoneal Dialysis for High-Risk Newborns after Cardiac Surgery

**DOI:** 10.34067/KID.0000000691

**Published:** 2025-01-08

**Authors:** Elvia Rivera-Figueroa, Md Abu Yusuf Ansari, Emily Turner Mallory, Padma Garg, Mary B. Taylor, Ali Mirza Onder

**Affiliations:** 1 Division of Pediatric Critical Care, Batson Children's Hospital of Mississippi, University of Mississippi, Jackson, Mississippi; 2 Division of Pediatric Critical Care, Puerto Rico Women's and Children's Hospital, Ponce Health Sciences University, Bayamon, Puerto Rico; 3 Department of Data Science, University of Mississippi Medical Center, Jackson, Mississippi; 4 Medical School, University of Mississippi, Jackson, Mississippi; 5 Division of Pediatric Nephrology, Batson Children's Hospital of Mississippi, University of Mississippi, Jackson, Mississippi; 6 Division of Pediatric Nephrology, Nemours Children's Health, Wilmington, Delaware

**Keywords:** congenital heart surgery, newborn, early peritoneal dialysis, urine output, fluid overload

## Abstract

**Key Points:**

- Inability to achieve negative fluid balance in postoperative 24 hours may be a reliable surrogate marker to start early peritoneal dialysis (PD) after cardiac surgery.
- Prolonged cardiopulmonary bypass and aorta cross-clamp duration may determine PD catheter placement in the operating room.
- The first postoperative 8 hours was indiscriminative for the decision to start early PD for these high-risk newborns.

**Background:**

The beneficial effect of peritoneal dialysis (PD) catheter placement after cardiopulmonary bypass (CPB) in young infants has been demonstrated. However, the indications to start early PD are not agreed upon.

**Methods:**

This retrospective single-center study was conducted to evaluate the performance of a clinical strategy for early PD start. PD catheters were placed in the operating room after CPB. Those with prolonged CPB times (>180 minutes), postoperative (postop) oligoanuria, and/or inability to achieve negative fluid balance in postop 24 hours were evaluated as high risk and selected for early PD (PD [+]) start. All PD (+) were started within the first postop 24 hours. Primary outcomes were 5% fluid accumulation at postop 48 hours and severe AKI on postop day (POD) 5.

**Results:**

There were 49 newborns. Twenty-nine newborns were early PD (+) starts, and 20 used the PD catheter as an abdominal drain (PD −). Baseline demographic data were similar. Both groups were oliguric during first postop 8 hours (*P* = 0.906). The early PD (+) group produced significantly less urine output during POD 1 (0.98 versus 3.02 ml/kg per hour; *P* = 0.001). At postop 48 hours, the early PD (+) group had a similar prevalence of 5% fluid accumulation as early PD (−): 5 (16.7%) versus 2 (7.41%), respectively (*P* = 0.427). Severe AKI incidence on POD 5 was similar between the groups (17.3% versus 5.0%; *P* = 0.204). Time to extubation was longer for the early PD (+) group compared with the PD (−) group: 10.0 days (7.0–16.0) versus 4.0 days (4.0–10.0), respectively (*P* = 0.017).

**Conclusions:**

Persistent oliguria and inability to achieve negative fluid balance during initial postop 24 hours may identify those newborns who will benefit from early PD. The first postop 8 hours was indiscriminative for this strategy. PD start may ameliorate the disadvantage for the designated group.

## Visual Abstract

**Figure:**
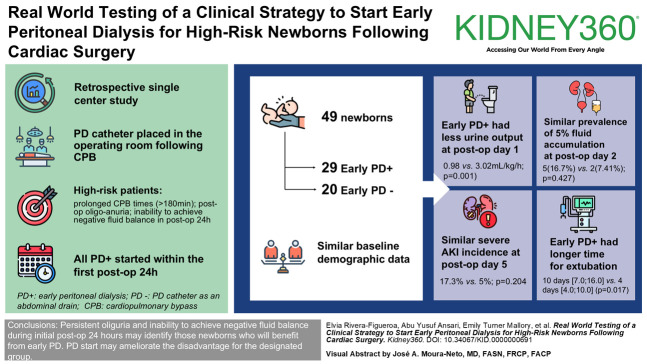

## Introduction

Cardiac surgery–associated AKI (CSA-AKI) is a frequently observed complication of surgical repair of complex congenital cardiac defects.^1,2^ The reported incidence of CSA-AKI is 15%–55%, with higher risk for newborns and young infants.^1,3,4^ In a recent multicenter large retrospective cohort, 9% of the newborns developed stage 3 AKI, which was independently associated with hospital mortality.^5^ Among newborns and young infants with CSA-AKI, KRT is used in 3%–5%, with fluid accumulation being the most common indication.^3,6^ The outcomes of these young children with CSA-AKI requiring KRT per cause remain poor and without much improvement over the years, with reported mortality being 55%–75%.^3,7,8^ This poor outcome may be partly related to worsening fluid accumulation due to delayed KRT start.^9^ Worsening fluid accumulation is demonstrated to be an independent predictor of unfavorable hospital outcomes for critically ill young children, with evidence showing that it is associated with increased in-hospital mortality.^9–11^

Pediatric and adult cardiac surgery continue to demonstrate improvement in morbidity/mortality with early initiation of KRT.^6,10–13^ There are no established protocols for prophylactic treatments to prevent AKI during cardiac surgery.^14,15^ Early postoperative (postop) fluid restriction and utilization of loop diuretics are the most commonly practiced methods of postop fluid balance management.^16^ For the past 30 years, a change of practice has evolved in some cardiac centers to place a peritoneal dialysis (PD) catheter after cardiopulmonary bypass (CPB) and start early PD or use the catheter as an abdominal drain.^10,12,17^ Early PD was used within the first postop 24–48 hours for better fluid management in patients at high risk of CSA-AKI.^10,17^ Moreover, the placement of PD catheter in the operating room (OR) after CPB was reported to produce more favorable outcomes for these young infants.^10,12,17,18^

We revised our cardiac intensive care unit (CICU) protocol in the beginning of 2012 in the Children's Hospital of Mississippi. High risk of postop severe AKI was determined as single-ventricle physiology palliations (Norwood/Sano), total anomalous pulmonary venous connection corrections, arterial switch operations, other palliations for complex congenital heart defects, CPB lasting longer than 180 minutes, and aorta cross-clamp time longer than 70 minutes.^19^ According to our new clinical strategy, all newborns and young infants who were at high risk of CSA-AKI had PD catheter placement after CPB while still in the OR. During the first postop 24 hours, newborns who remained oliguric despite intravenous (IV) fluid boluses and furosemide challenge and/or were unable to achieve negative fluid balance were considered to be at high risk of pathologic fluid accumulation and were initiated on PD, or early PD (+). For the remaining newborns, the PD catheters were used as abdominal drains, or early PD (−). In this retrospective study, we aimed (*1*) to evaluate the performance of the clinical decision strategy using the above indications for early PD start for those newborns with higher postop acuity and more risk factors because they were predicted to develop severe AKI and pathologic fluid accumulation, according to historical research data,^19^ and (*2*) to understand the differences in clinical and metabolic outcomes of newborns after cardiac surgery to postop day (POD) 5 between those who received early PD versus those who did not.

## Methods

### Sample Review

This is a retrospective chart review from a single, free-standing, tertiary care children's hospital affiliated with the University of Mississippi Medical Center, Jackson, Mississippi. The University of Mississippi Medical Science Campus Institutional Review Board approved the study protocol (Institutional Review Board: 2021-0628). After obtaining approval, a retrospective chart review was performed from the CICU database. Medical and surgical data were collected on 49 newborns, all younger than 4 weeks with PD catheter placement in the OR after congenital heart surgery between January 1, 2012, and December 31, 2015. Clinical investigators entered the data into REDCap, and a statistician received the study data through the REDCap format in a deidentified form to ensure health record confidentiality.^20,21^ Clinical outcomes were followed and analyzed through the first postop 5 days.

Clinical, demographic, and surgical data are summarized in Table 1. Comparative clinical and laboratory values are listed in Table 2. Gestational age information was not available for all infants and thus was not collected nor included in the statistical analysis.

**Table 1:** Baseline demographic, laboratory, and clinical data for the early PD (+) and PD (−) groups

Demographic and Clinical Characteristics	All, *N*=49	Early PD (+), *N*=29	Early PD (−), *N*=20	*P* Value
Male, No. (%)	29 (59.2)	15 (51.7)	14 (70.0)	0.325
Age at surgery, d	7.00 (4.00–9.00)	6.00 (4.00–8.00)	7.50(4.75–14.00)	0.187
Newborn	49	29	20	NA
Height, cm	48.0 (47.0–50.0)	49.0 (47.0–51.0)	48.0 (45.0–49.0)	0.594
Weight, kg	3.10 (2.80–3.40)	3.02 (2.80–3.40)	3.23 (2.81–3.40)	0.515
**RACHS-1 score, No. (%)**				0.352
2	2 (4.08)	0 (0.00)	2 (10.0)	
3	11 (22.4)	8 (26.7)	3 (15.0)	
4	11 (22.4)	7 (24.1)	4 (20.0)	
6	25 (51.0)	14 (48.3)	11 (55.0)	
Single-ventricle physiology–yes	30 (61.2)	18 (62.1)	12 (60.0)	0.999
Preoperative serum creatinine, mg/dl	0.49 (0.38–0.65)	0.53 (0.38–0.73)	0.46 (0.39–0.57)	0.207
Preoperative BUN, mg/dl	13.0 (6.0–17.0)	11.0 (6.0–17.0)	14.5 (10.0–23.0)	0.172
Preoperative AKI; yes, No. (%)	4 (8.16)	2 (6.90)	2 (10.0)	0.999
Preoperative fluid overload, %	15.2 (7.6–27.1)	14.4 (7.3–26.9)	18.5 (11.1–29.1)	0.393
**Surgical procedures, No. (%)**				0.716
Arterial switch	5 (10.2)	3 (10.3)	2 (10.0)	
Norwood/Sano	23 (46.9)	12 (41.4)	11 (55.0)	
Other	11 (22.4)	6 (20.7)	5 (25.0)	
PDA ligation	5 (10.2)	4 (13.8)	1 (5.0)	
TAPVR repair	5 (10.2)	4 (13.8)	1 (5.0)	
CPB time, min	180.0 (146.0–222.0)	197.0 (154.0–235.0)	155.0 (141.0–180.0)	0.027^a^
Aorta cross-clamp time, min	71.0 (46.0–102.0)	90.0 (58.0–113.0)	53.5 (45.0–68.2)	0.021^a^

Differences between the categorical data were tested using Chi-squared test (or Fisher's exact test when the cell counts are below 5). All continuous data were presented as median (first quartile–third quartile), and their differences between the groups were tested using Mann–Whitney *U* test. CPB, cardiopulmonary bypass; PD, peritoneal dialysis; PDA, patent ductus arteriosus; RACHS, risk adjusted classification for congenital heart surgery; TAPVR, total anomalous pulmonary venous return.

a *P* values signify significant association at a 0.05 level of significance.

**Table 2:** Preoperative and immediately postop laboratory and hemodynamic predictors

Predictors of Outcome	All, *N*=49	Early PD (+), *N*=29	Early PD (−) *N*=20	*P* Value
**Preoperative predictors**				
Hct, %	37.0 (34.0–39.0)	36.3 (35.0–38.0)	38.0 (33.5–41.5)	0.258
Albumin, g/dl	2.40 (2.15–2.75)	2.60 (2.40–2.80)	2.30 (2.12–2.63)	0.552
Serum pH	7.40 (7.36–7.44)	7.40 (7.36–7.44)	7.38 (7.36–7.43)	0.653
Serum lactate	1.20 (1.00–1.75)	1.40 (1.00–1.85)	1.10 (0.88–1.25)	0.213
Sys BP, mm Hg	68.0 (58.5–76.0)	68.0 (57.0–76.0)	67.5 (60.8–76.0)	0.974
Dias BP, mm Hg	37.0 (32.0–43.0)	37.0 (31.5–41.5)	38.5 (32.8–44.0)	0.457
MAP, mm Hg	47.0 (43.2–53.8)	47.0 (41.0–53.2)	48.5 (44.8–54.0)	0.340
**Immediate postop predictors**				
Serum creatinine, mg/dl	0.47 (0.38–0.58)	0.48 (0.39–0.60)	0.46 (0.37–0.53)	0.218
BUN, mg/dl	10.0 (6.0–12.0)	8.0 (6.0–12.0)	12.0 (9.75–16.0)	0.036^a^
Hct, %	43.0 (38.0–47.0)	44.4 (37.0–47.0)	41.5 (39.8–47.2)	0.706
Albumin, g/dl	2.45 (2.38–2.67)	2.45 (2.38–2.67)	NA	NA
pH	7.38 (7.31–7.44)	7.36 (7.31–7.41)	7.39 (7.34–7.45)	0.328
Serum lactate	3.70 (2.60–4.80)	3.30 (3.00–5.30)	4.05 (2.30–4.55)	0.714
CVP, mm Hg	8.0 (7.0–10.0)	9.0 (7.5–11.0)	8.0 (5.5–8.75)	0.103
Sys BP, mm Hg	59.0 (57.0–76.0)	59.0 (55.0–69.0)	61.5 (59.0–85.5)	0.074
Dias BP, mm Hg	37.0 (31.5–44.0)	36.0 (31.0–41.0)	40.5 (36.2–46.2)	0.054
MAP, mm Hg	45.0 (42.0–52.0)	44.0 (39.0–49.0)	50.0 (45.0–60.8)	0.002^a^
**POD 1 predictors**				
Serum creatinine, mg/dl	0.57 (0.35–0.79)	0.68 (0.56–0.85)	0.58 (0.51–0.69)	0.047^a^
BUN, mg/dl	16.0 (11.0–20.0)	14.0 (11.0–19.0)	17.5 (12.8–22.0)	0.148
Hct, %	42.0 (36.0–47.0)	41.6 (36.0–44.0)	42.5 (38.0–46.0)	0.859
pH	7.41 (7.37–7.46)	7.40 (7.35–7.45)	7.42 (7.41–7.47)	0.041^a^
Serum lactate	2.40 (1.70–3.60)	2.80 (2.00–3.80)	1.95 (1.40–3.37)	0.075
CVP, mm Hg	9.0 (8.0–11.0)	9.0 (7.5–10.2)	9.0 (8.0–11.0)	0.795
Sys BP, mm Hg	59.5 (52.0–66.8)	58.0 (51.2–62.8)	64.5 (53.8–72.8)	0.105
Dias BP, mm Hg	37.0 (33.8–42.0)	36.0 (28.5–39.8)	37.0 (35.0–43.5)	0.165
MAP, mm Hg	44.0 (39.0–49.0)	42.0 (37.0–45.0)	46.0 (42.8–55.5)	0.007

Differences between the categorical data were tested using Chi-squared test (or Fisher's exact test when the cell counts are below 5). All continuous data were presented as median (first quartile–third quartile), and their differences between the groups were tested using Mann–Whitney *U* test. CVP, central venous pressures; Dias, diastolic; Hct, hematocrit; MAP, mean arterial pressure; NA, not available; PD, peritoneal dialysis; POD, postoperative day; postop, postoperative; Sys, systolic.

a *P* values signify significant association at a 0.05 level of significance.

### Surgery and Postop Period

**Figure 1:**
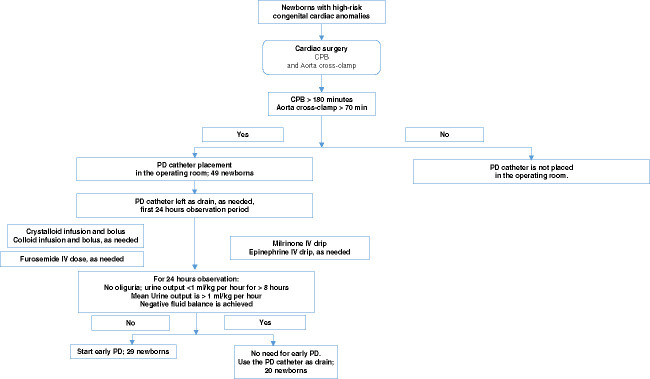
**Flow chart for the clinical strategy to start early PD.** Newborns with high-risk congenital cardiac anomalies were identified. Those with CPB duration >180 minutes and aorta cross-clamp time >70 minutes received PD catheters in the OR. All newborns are observed for up to 24 hours after surgery for urine output and ability to achieve negative fluid balance. Those who demonstrated suboptimal response to IV fluid replacement and furosemide administration were started on early PD. For the others, the PD catheter was used as an abdominal drain, when indicated. CPB, cardiopulmonary bypass; IV, intravenous; OR, operating room; PD, peritoneal dialysis.

During the full duration of the study period, a single pediatric cardiothoracic surgeon performed all the surgeries. The cardiothoracic anesthesia team and the certified clinical perfusionist team were the same throughout the study period. All newborns in the study underwent surgical palliation of their lesions using CPB and/or with clamping of the aorta. Hypothermia was used when needed and hypothermic circulatory arrest was used in newborns with single-ventricle diagnosis undergoing first- and second-stage repairs. After the completion of cardiac surgery and disconnection from CPB, a Dacron single-cuffed straight silicone rubber acute PD catheter (Tenckhoff catheter, Cook Medical Inc., Bloomington, IN) was inserted in the abdomen under general anesthesia below the umbilicus and clamped to drainage. Figure 1 demonstrates the flow chart of the clinical decision-making strategy used in this study.

A urinary catheter was inserted into the bladder in the OR and remained during the 48- to 72-hour postop period for all patients. Central venous pressures were measured using a central venous catheter, and systemic arterial pressures were continuously measured in the radial or femoral artery using an invasive arterial catheter. IV fluid boluses were used to maintain central venous pressures and to rescue arterial hypotension episodes. Plasma-Lyte (Baxter Healthcare Corporation, Deerfield, IL), 0.9% normal saline, and albumin 5% were the main IV solutions used. As per unit protocol, the newborns were placed on milrinone infusion for afterload reduction and low-dose epinephrine infusion for cardiac contractility in the OR before arrival to the unit. Calcium chloride IV boluses and infusions were commonly used to improve cardiac function. Severe AKI was defined as AKI stage 2 or stage 3 using the neonatal modified Kidney Disease Improving Global Outcomes criteria, with the maximum criteria fulfilled either as change in serum creatinine or duration of oligoanuria.

### Early PD

Clinical indications that prompted to start early PD were prolonged CPB time (>180 minutes), prolonged aorta cross-clamp time (>70 minutes), postop oliguria (<1 ml/kg per hour) for more than 8 hours unresponsive to fluid administration and to furosemide 1 mg/kg per dose intravenous challenge within the first postop 24 hours, and/or inability to achieve negative fluid balance in the first postop 24 hours.^19^ The ultimate decision to start PD was made by the lead cardiothoracic surgeon in conjunction with the CICU attending physician on a case-by-case basis using the revised clinical strategy described above. Once PD was initiated, these newborns were not treated with furosemide or chlorothiazide during the period of PD treatment.

Manual continuous cyclic PD was used, and the treatment was performed by the bedside intensive care unit nurses. The start PD prescription was a fill volume of 10 ml/kg and 1.5% dextrose dialysis fluid (Dianeal, Baxter Healthcare, McGaw Park, IL) with 45-minute fill-and-dwell times and 15-minute drain time. The treatment was 24-hour continuous dialysis, as tolerated. A closed-circuit system was used for all PD treatments. These PD circuits were set up by dialysis nurses every 72 hours. In those newborns for whom PD was not initiated, the catheters were left open for passive drainage, as clinically indicated and as tolerated.

### Statistical Analyses

Categorical variables were summarized by relative frequencies and expressed as percentages. Chi-squared test was applied to assess differences between the two PD groups. When expected cell counts were less than five, Fischer's exact test was used instead because of its appropriateness for smaller sample sizes.

Continuous variables were reported using median and interquartile range (IQR), with the IQR denoted as (first quartile–third quartile). Differences between groups for these variables were evaluated using Mann–Whitney *U* test, suitable for skewed data distribution. All analyses were performed using R Statistical Software (v4.2.1; R Core Team 2022).^22^

## Results

There were 49 newborns in the study. Postop early PD was initiated in 29 of the newborns (early PD [+]), and for 20 newborns, PD catheters were used as abdominal drains when indicated (early PD [−]). There were 29 male newborns (59.2%), and the median age at surgery was 7 days (IOR, 4–9 days). Gestational age was not included as one of the predictor variables. Table 1 summarizes the demographic data. All early PD (+) newborns were started on PD during the first postop 24 hours and dialyzed for 48–72 hours afterward. None of the early PD (−) group newborns were started on PD during the first postop 5 days of this study.

The comparative demographic and surgery data are summarized in Table 1. The two cohorts had similar preoperative serum creatinine and BUN values, comparable preoperative AKI incidence, and comparable preoperative fluid accumulation percentages. The early PD (+) group had longer CPB time (197.0 minutes [154.0–235.0] versus 155 minutes [141.0–180.0]; *P* = 0.027) and aorta cross-clamp time (90.0 minutes [58.0–113.0] versus 53.5 minutes [45.0–68.2]; *P* = 0.021). Table 1 summarizes these findings for both groups.

The comparative clinical and laboratory findings are detailed in Table 2. Immediate postop BUN was lower for the early PD (+) group (*P* = 0.036). Similarly, immediate postop mean arterial pressure was recorded to be lower for the early PD (+) group (*P* = 0.002).

### Fluid Balance and 5% Fluid Accumulation

**Figure 2:**
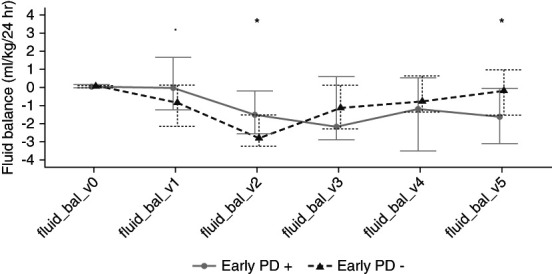
**Comparative daily fluid balance (ml/kg per 24 hour) for the postop 5 days, stratified according to early PD (+) versus early PD (−).** *P < 0.05. postop, postoperative.

Both groups achieved a progressive negative fluid balance in the first postop 3 days. Fluid balance was positive during the first postop 8 hours for both groups, the early PD (−) group being 1.60 ml/kg per 24 hours (−0.81 to 2.49) and the early PD (+) group being 1.21 ml/kg per 24 hours (−0.40 to 3.88) (*P* = 0.631). On POD 1, the early PD (−) group achieved −20 ml/kg per 24 hours (−51.51 to 3.11) fluid balance compared with the early PD (+) group achieving −0.64 ml/kg per 24 hours (−29.76 to 39.8) (*P* = 0.090). Figure 2 details these findings and the statistical comparison. Table 3 presents the comparative fluid balance values between the two PD groups.

**Table 3:** Comparative fluid balance data (ml/kg per 24 hour) according to the PD groups for the first postoperative 5 days

POD	Early PD (+), *N*=29	Early PD (−), *N*=20	*P* Value
POD 0	1.21 (−0.40 to 3.88)	1.60 (−0.81 to 2.49)	0.631
POD 1	−0.64 (−29.76 to 39.8)	−20.10 (−51.51 to 3.11)	0.090
POD 2	−36.27 (−61.35 to −4.57)	−67.32 (−77.9 to −36.69)	0.031^a^
POD 3	−52.01 (−69.37 to 14.6)	−26.92 (−54.80 to 2.80)	0.490
POD 4	−28.52 (−83.96 to 12.8)	−18.38 (−32.73 to 15.1)	0.326
POD 5	−38.91 (−74.40 to −1.18)	−4.21 (−36.6 to 23.5)	0.013^a^

These data are demonstrated in the article in Figure 1. All continuous data were presented as median (first quartile–third quartile), and their differences between the groups were tested using Mann–Whitney *U* test. PD, peritoneal dialysis; POD, postoperative day.

a *P* values signify significant association at a 0.05 level of significance.

At postop 48 hours, the early PD (+) group had five newborns (16.7%) with 5% fluid accumulation while the early PD (−) group had two (7.41%) (*P* = 0.427, Supplemental Table 1).

### Urine Output

**Figure 3:**
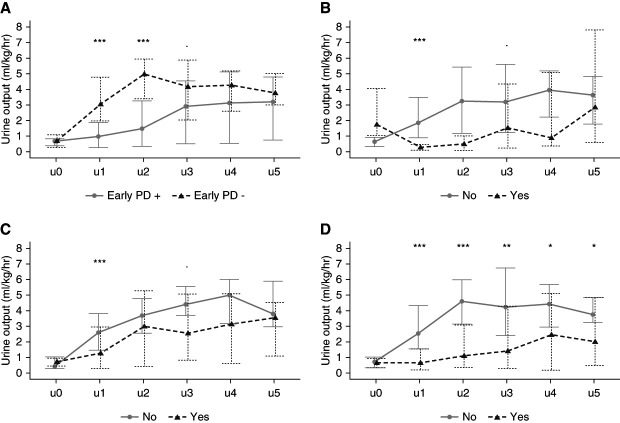
**Comparative urine outcomes for the first postop 5 days, including the immediate postop 8 hours (u0).** (A) Early PD (+) versus early PD (−). (B) 5% fluid accumulation at postop 48 hours versus <5% fluid accumulation. (C) Severe AKI on postop 48 hours versus no severe AKI. (D) Severe AKI on postop day 5 versus no severe AKI. *P < 0.1, **P < 0.05, ***P<0.001.

Urine output (ml/kg per hour) was similar for the two groups during the first 8 hours after arriving to the CICU (early PD (+) 0.67 (0.38–0.84) versus early PD (−) 0.66 (0.26–1.10) ml/kg per hour; *P* = 0.906)). The urine output increased for POD 1 for both groups, and the early PD (+) group produced significantly less urine output compared with early PD (−): 0.98 (0.26–1.87) versus 3.02 (1.98–4.76) ml/kg per hour, respectively (*P* = 0.001). There was persistent increase in urine output for both groups on POD 2 (early PD (+) 1.48 (0.34–3.25) versus early PD (−) 4.95 (3.40–5.92) ml/kg per hour, respectively; *P* < 0.001). Table 4 presents the comparative urine output values with IQR for each of the PODs. Figure 3 displays these findings.

**Table 4:** Comparison of the clinical and laboratory outcome variables between early PD (+) versus PD (−) newborns

Comparative Outcomes	All, *N*=49	Early PD (+), *N*=29	Early PD (−), *N*=20	*P* Value
Urine output at postop 8 h	0.67 (0.34–1.01)	0.67 (0.38–0.84)	0.66 (0.26–1.10)	0.906
Urine output on day 1	1.74 (0.57–3.35)	0.98 (0.26–1.87)	3.02 (1.98–4.76)	0.001^a^
Urine output on day 2	3.16 (1.01–5.14)	1.48 (0.34–3.25)	4.95 (3.40–5.92)	<0.001^a^
Urine output on day 3	2.98 (1.18–5.34)	2.90 (0.51–4.54)	4.18 (2.03–5.86)	0.099
Urine output on day 4	3.88 (1.69–5.18)	3.12 (0.54–5.11)	4.24 (2.59–5.18)	0.215
Urine output on day 5	3.59 (1.56–4.93)	3.19 (0.73–4.79)	3.79 (3.01–5.01)	0.259
Fluid balance at postop 8 hours	330 (106–905)	480 (114–963)	215 (92.6–822)	0.371
Fluid balance on day 1	−47.9 (−156.6 to 29.4)	−14.1 (−85.6 to 29.5)	−109.85 (−195.7 to 17.1)	0.088
Fluid balance on day 2	−145.70 (−240.50 to −64.40)	−39.56 (−126.3 to 6.4)	−124.85 (−145.2 to 68.5)	0.094
5% fluid overload at postop 8 h, No. (%)	2 (4.10)	2 (6.90)	0 (0.0)	0.507
5% fluid overload on day 1, No. (%)	6 (12.2)	4 (13.8)	2 (10.0)	0.99
5% fluid overload on day 2, No. (%)	5 (10.2)	4 (13.8)	1 (5.0)	0.636
10% fluid overload on day 2, No.(%)	4 (8.16)	4 (13.8)	0 (0.0)	0.135
**AKI at postop 48 h, No. (%)**				0.498
No AKI/mild AKI	35 (71.4)	19 (65.5)	16 (80.0)	
Severe AKI	14 (28.6)	10 (34.5)	4 (20.0)	
**AKI at postop day 5**				0.204
No AKI/mild AKI, No. (%)	43 (87.8)	24 (82.7)	19 (95.0)	
Severe AKI, No. (%)	6 (12.2)	5 (17.3)	1 (5.0)	
Time to extubation, d	7.5 (4.0–11.2)	10.0 (7.0–16.0)	4.0 (4.0–10.0)	0.017^a^
Time to chest closure, d	3.0 (2.0–5.0)	4.0 (2.0–6.0)	2.5 (2.0–4.0)	0.067
CICU stay, d	20.0 (16.0–37.00)	21.0 (16.0–31.2)	19.0 (12.0–40.0)	0.572

Differences between the categorical data were tested using Chi-squared test (or Fisher's exact test when the cell counts are below 5). All continuous data were presented as median (first quartile–third quartile), and their differences between the groups were tested using Mann–Whitney *U* test. CICU, Cardiac Intensive Care Unit; PD, peritoneal dialysis; postop, postoperative.

a *P* values signify significant association at a 0.05 level of significance.

### Serum Creatinine and AKI Trajectories

**Figure 4:**
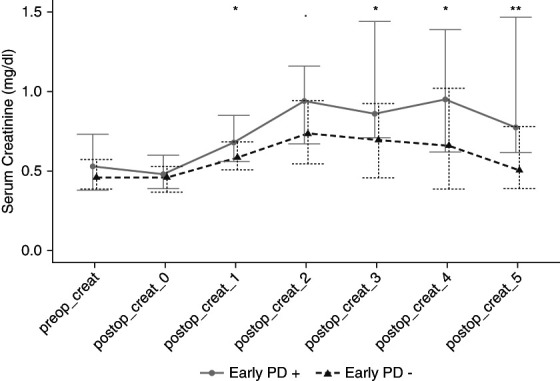
**Comparative serum creatinine trajectories with preoperative, immediate postop, and postop trajectory values, stratified according to early PD (+) and early PD (−) groups.** **P < 0.01, *P < 0.05, *P* < 0.1.

Both preoperative serum creatinine and immediate postop serum creatinine were similar for the two groups. The serum creatinine trajectory separated on POD 1 because of increasing serum creatinine for the early PD (+) group, reaching statistical difference (*P* = 0.047). Figure 4 demonstrates the serum creatinine trajectories for the two groups. Supplemental Table 2 summarizes the comparative serum creatinine data.

At postop 48 hours, the early PD (+) group tended to have more newborns with severe AKI when compared with the early PD (−) group: 10 (34.5%) versus 4 (20%), *P* = 0.498, respectively. On POD 5, the two groups demonstrated statistically similar severe AKI incidence: 5 (17.3%) versus 1 (5.0%), *P* = 0.204, respectively. When defining severe AKI, 16 of 20 newborns (80%) were assigned to their AKI stage according to the highest change in serum creatinine value and for 4 of 20 (20%), lowest urine output criteria were used.

The urine outputs of newborns with severe AKI on POD 5 were similar to those who were AKI free for the immediate postop 8 hours because both groups were oliguric. The trajectories diverged starting on POD 1 (*P* < 0.001). These are presented in Figure 3D and Supplemental Table 3.

### Other Clinical Outcomes and Complications

**Figure 5:**
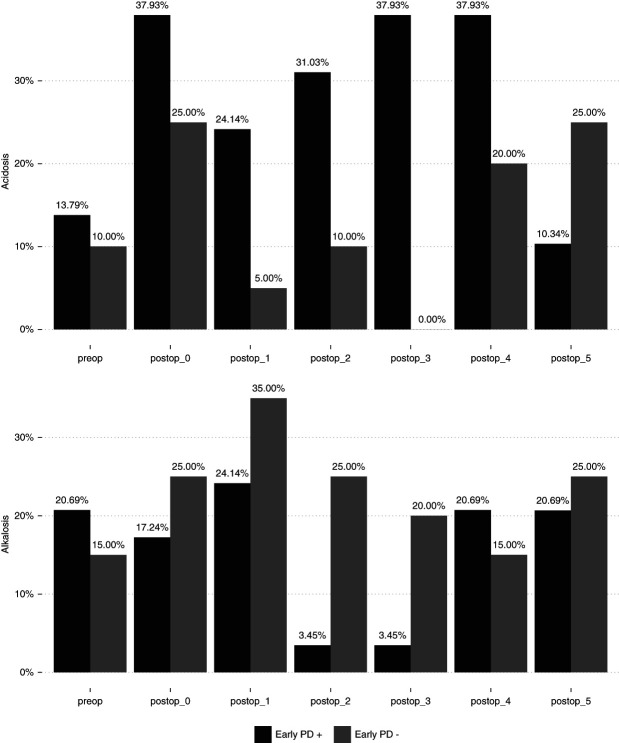
**Comparative incidence of metabolic acidosis and metabolic alkalosis stratified according to early PD (+) versus early PD (−) groups for the postop 5 days.** Metabolic acidosis was more likely for the early PD (+) group starting on POD 1 until POD 4. POD, postoperative day.

There was no statistical difference between the two groups when evaluated for CICU stay (*P* = 0.572). The early PD (+) group trended to later chest closure: 4.0 days (2.0–6.0) versus 2.5 days (2.0–4.0) (*P* = 0.067), respectively. Similarly, the early PD (+) cohort demonstrated longer time to extubation: 10.0 (7.0–16.0) versus 4.0 (4.0–10.0); (*P* = 0.017), respectively. Table 4 summarizes these findings. Metabolic acidosis was more likely for the early PD (+) group starting on POD 1 until POD 4. Figure 5 demonstrates the incidence of metabolic acidosis and metabolic alkalosis during the study period for both groups.

There were no deaths among the study participants during the study observation period. There were no major complications that resulted in discontinuation of PD in the early PD (+) group. All PD catheters in the early PD (−) cohort were used as peritoneal drains, with large variation of durations, during the study period, with median use of 2.5 days (0.5–4.5).

### Multinomial Logistic Regression Analysis

We performed multinomial logistic regression analysis for the two primary outcome variables 5% fluid accumulation at 48 hours and severe AKI on POD 5. All the previously determined predictors were included in the initial analysis. When compared with the early PD (−) group, the early PD (+) group was 3.18 times more likely to present with severe AKI on POD 5. None of the other predictors, age at surgery, CPB duration, and aorta cross-clamp duration increased the likelihood of this outcome. These findings are summarized in Table 5.

**Table 5:** Association between AKI on day 5 and relevant characteristics of the newborn babies using multinomial logistic regression

Predictor	Mild AKI			Severe AKI		
	aOR	95% CI	*P* Value	aOR	95% CI	*P* Value
**PD group**						
Early PD (−)	Ref.			Ref.		
Early PD (+)	1.48	0.34 to 6.43	0.595	4.18	0.36 to 48.37	0.244
Age at surgery, days	0.97	0.87 to 1.07	0.492	0.96	0.84 to 1.10	0.560
Aorta cross-clamp duration	1.01	0.99 to 1.03	0.453	0.99	0.96 to 1.02	0.476
CPB duration	1.00	0.99 to 1.02	0.533	1.01	0.99 to 1.03	0.157

No AKI was the reference group in the response. The sample size was 49. aOR, adjusted odds ratio; CI, confidence interval; CPB, cardiopulmonary bypass; PD, peritoneal dialysis.

The early PD (+) group was 0.77 times more likely to present with 5% fluid accumulation at 48 hours when the early PD (−) group was taken as reference. The other predictors, age at surgery, aorta cross-clamp duration, and CPB duration were insignificant for this outcome. These findings are summarized in Table 6.

**Table 6:** Association between 5% fluid overload at 48 hours and relevant characteristics of the newborn babies using binary logistic regression

Predictor	aOR	95% CI	*P* Value
**PD group**			
Early PD (−)	Ref.		
Early PD (+)	1.77	0.26 to 11.90	0.583
Age at surgery, days	0.93	0.77 to 1.13	0.499
Aorta cross-clamp duration	1.03	1.00 to 1.06	0.081
CPB duration	0.98	0.96 to 1.00	0.075

The sample size was 49. aOR, adjusted odds ratio; CI, confidence interval; CPB, cardiopulmonary bypass; PD, peritoneal dialysis.

Further analysis was performed for validation of the predictors used in the clinical strategy. For that purpose, binary logistic regression was performed to evaluate the association between the commonly used indications of early PD start and the PD groups (early PD [+] versus early PD [−]). Urine output on POD 1 was statistically significant and demonstrated an inverse association for predicting early PD (+) with an adjusted odds ratio of 0.68 (0.44 to 0.96, *P* = 0.026). CPB duration was significant in the bivariate analysis (*P* = 0.027). This statistical significance was lost when CPB duration predictor was analyzed with urine output on POD 1 data using binary logistic regression (adjusted odds ratio, 1.01 [1.00 to 1.02], *P* = 0.06). These findings are summarized in Table 7.

**Table 7:** Association between peritoneal dialysis groups (early peritoneal dialysis + versus early peritoneal dialysis −) and relevant characteristics of the newborn babies using binary logistic regression

Predictor	aOR	95% CI	*P* Value
Urine output on POD 1	0.68	0.44 to 0.96	0.026
Cardiopulmonary bypass duration	1.01	1.00 to 1.02	0.06

Early peritoneal dialysis − was the reference group in the response. The sample size was 49. aOR, adjusted odds ratio; CI, confidence interval; POD, postoperative day; postop, postoperative.

## Discussion

In this retrospective analysis, we evaluated the performance of a clinical strategy in deciding which newborns should have early PD start after cardiac surgery. Approximately two thirds of the newborns classified as high risk perioperatively were started on early PD. Compared with the group not started on early PD, the early PD (+) group achieved negative fluid balance with 24-hour delay and maintained the negative fluid balance primarily because of ultrafiltration rather than urine output. The early PD (+) group also showed a trend toward higher incidence of 5% fluid accumulation at postop 48 hours and higher incidence of severe AKI on POD 5, although these differences did not reach statistical significance. Finally, the early PD (+) group took longer time to extubation. All these findings suggest that the early PD (+) group had higher postop acuity and more risk factors of severe AKI and pathologic fluid accumulation. This clinical decision-making strategy performed well in identifying those who should start early PD because none of the newborns from the early PD (−) cohort required PD during the 5-day postop period. These findings further suggest that preoperative and intraoperative risk factors can effectively guide the decision to place a PD catheter in the OR. Furthermore, the persistent postop oligoanuria and the inability to achieve negative fluid balance within the postop 24 hours may serve as an additional criterion for identifying newborns who may need early PD initiation in the initial postop 24 hours.

The two study groups were comparable for the preoperative demographic, clinical, and laboratory data. Longer CPB and aorta cross-clamp times are well described as risk factors of CSA-AKI.^2,8,23^ The immediate postop mean arterial pressure was significantly lower for the early PD (+) group.^24,25^ The BUN at the same time being lower for the early PD (+) group may be a surrogate marker for worse heart function, leading to fluid overload and intravascular dilution. Nonetheless, the main narrative is probably written by urine output. Despite urine output being similar between the two groups during the first postop 8 hours and both groups being oliguric, the brisk response to furosemide for the early PD (−) group during postop 24 hours was instrumental in differentiating those who may not need early PD start. These findings help us conclude on two points; the preoperative and intraoperative data may help to identify newborns who will likely benefit from getting the PD catheter placement in the OR. However, for early PD start, one should observe the urine output and the response to furosemide in the first postop 24 hours.^26,27^ Our single-center data suggest that despite a similar preoperative CSA-AKI risk profile, early PD may not be needed for all high-risk newborns after cardiac surgery.

The daily data defining the trajectory of postop fluid balance for both PD groups was very informative. The early PD (+) group's negative fluid balance was predominantly due to their PD ultrafiltrate, suggesting the irreplaceable benefit of early PD start for this group. The early PD (+) group demonstrated significantly less daily urine output compared with the early PD (−) group. Having said that, these data should not be interpreted as the comparative success of diuretic management versus PD because, as stated above, the clinical data suggest that the early PD (+) group endured more severe AKI and demonstrated more postop clinical acuity. These newborns would likely have taken longer to achieve the desired negative fluid balance in the absence of early PD start.

Our work reiterates the shortcomings of the use of serum creatinine to identify those at high risk of CSA-AKI and fluid accumulation early during postop care.^28–30^ When those 14 newborns with severe AKI at 48 hours were investigated after postop 24 hours, 12 fulfilled the urinary diagnostic criteria, compared with only three fulfilling the serum creatinine criteria.^26,27^ Therefore, serum creatinine may truly be suboptimal in the first 24–48 hours for identifying newborns at high risk of fluid overload.^30–33^ It may even be paradoxical that worse the kidney injury and worse the fluid accumulation, the dilution effect on serum creatinine may further mask the underlying kidney injury.^31–33^ These findings further support the use of early urine output as a surrogate biomarker for severe AKI and fluid accumulation during the early postop state.^27,34–36^

As always, there are several limitations to our study. Most importantly, this study reports the results of a retrospective cohort without randomization. The decisions to place the PD catheter in the OR and to start PD early were made on clinical grounds according to risk factors determined by the historical research data and demonstrated in the preoperative and surgical periods and according to urine output during the postop 24 hours, respectively. Moreover, because our study period is about a decade old, there may be some subtle upgrades for the standard postop care of these newborns, making some of the study findings less applicable to CICUs at present. At the same time, we also have strengths for our study. The cardiac surgeon and the CICU team were the same physicians throughout the study, making it more consistent care. All newborns considered to be at high risk of CSA-AKI received the Tenckhoff catheter in the OR. All PDs were initiated on those with poor urine response in the first postop 24 hours, and if PD was not initiated early, none of these newborns received PD for the postop 5 days, showing consistency in clinical practice during the study period. The laboratory, clinical, and surgical predictors and data used for this study are considered the standard of care and routinely available in most CICUs across the country. This may allow our findings to be applicable to newborns in other centers without having to diverge significantly from daily practice.

In conclusion, we demonstrated that oligoanuric state and inability to achieve negative fluid balance with furosemide challenge in the initial postop 24 hours may be reliable surrogate markers to detect newborns who will benefit from early PD start after cardiac surgery. High-risk congenital heart defect, prolonged CPB duration, and longer aorta cross-clamp duration may be useful to identify those who should get the PD catheter in the OR. However, more than one third of these high-risk newborns safely did not require early PD, and therefore, the diligent observation of their urine response during the initial postop 24 hours may be irreplaceable in making the decision. Having the PD catheter placed in the OR may still be advantageous. These findings bring hope for precision medicine in the postop care of these high-risk newborns and should promote prospective trials to better define those in need of early postop PD.

## Data Availability

Previously published data were used for this study. All data are included in the manuscript and/or supporting information. *Cardiology in the Young*, 1–8, doi:10.1017/S1047951123004286.

## Supplemental Material

Supplemental Methods

Supplemental Table 1. Comparative incidence of 5% fluid overload for the first postop 2 days according to the PD groups.

Supplemental Table 2. Comparative serum creatinine trajectories, starting with preoperative serum creatinine and daily postop serum creatinine according to the PD groups for the first postop 5 days. These data are demonstrated in the article in Figure 4.

Supplemental Table 3. Comparative urine outputs for the first postop 5 days segregated according to the outcome “severe AKI on postop day 5.” These data are demonstrated in Figure 3D.
